# Load matters: squat and deadlift mechanics near maximal effort

**DOI:** 10.3389/fspor.2026.1960762

**Published:** 2026-09-23

**Authors:** Michael Hales

**Affiliations:** Kennesaw State University, Kennesaw, GA, United States

**Keywords:** biomechanics, deadlift, load intensity, maximal strength, mechanical constraints, squat

## Introduction

The squat and deadlift are widely considered foundational resistance exercises and are frequently grouped together due to their shared involvement of the hip and knee extensors (1–3). This similarity has contributed to the common assumption that improvements in one lift will transfer directly to the other.

Much of the existing biomechanical literature supports this view, particularly when analyses are conducted under submaximal loading conditions (1–4). Previous studies examining squat and deadlift mechanics have typically been conducted at submaximal intensities, where coordinated joint motion and stable trunk posture can be maintained (1–3). Under these conditions, both lifts often demonstrate similar joint contributions and muscle activation patterns (1, 2, 5).

However, less attention has been given to how movement strategy may change as loads approach maximal effort. Recent work examining lifting biomechanics at higher intensities suggests that kinematic and kinetic variables change as load approaches maximal effort (4, 6), indicating that movement strategies may not scale linearly from submaximal conditions (4, 7, 8). As force demands increase, mechanical constraints may influence how movement is organized, potentially revealing differences between lifts that are less apparent at moderate intensities.

Recent computational modeling provides additional theoretical support for this possibility. Joint-moment-limited models of the squat (9, 10) demonstrated that increasing external load can produce changes in posture and the distribution of mechanical demand across joints rather than simply scaling the movement pattern observed at lower loads. Although computational models do not provide direct evidence of movement behavior during maximal lifting, these findings illustrate how increasing mechanical constraints may contribute to load-dependent changes in movement strategy. The importance of mechanical constraints in shaping coordination strategies has also been demonstrated in other multi-joint movements, including maximal vertical jumping (11).

While prior studies have documented biomechanical similarities and differences between the squat and deadlift, the present perspective focuses on how these relationships may be expressed under maximal loading conditions, drawing from previously published observational data and existing mechanical principles rather than new experimental manipulation or analysis.

## Observations from maximal lifting conditions

The observations discussed in this perspective are drawn from previously published motion capture data collected during a sanctioned powerlifting competition (6). No unpublished data or new analyses of the original dataset are included in the current manuscript. Rather, previously reported observations are reconsidered here in the context of existing biomechanical evidence and mechanical principles relevant to lifting as loads approach maximal effort. Unlike laboratory-based studies, these observations reflect performance under conditions where force-production demands approach the lifter's maximal capacity. The original analysis involved a limited, non-generalizable sample of lifters performing maximal attempts under competition conditions; accordingly, the observations considered here should be interpreted as exploratory. In order to reduce variability in stance mechanics, the original analysis included only lifters who performed both a squat with a stance no wider than shoulder width and a conventional deadlift (6). While this restriction limited the number of eligible participants, it allowed for comparison of movement strategies within the same individuals.

The research group reported distinct kinematic differences between the squat and conventional deadlift during competition-level attempts (6). The squat demonstrated a relatively coordinated pattern of hip and knee extension through the sticking region, whereas the deadlift exhibited a more sequential pattern in which joint contributions shifted throughout the ascent. These previously reported observations are descriptive in nature; the interpretations that follow are intended to consider these differences within a mechanical framework.

## Mechanical constraints and movement strategy

These differences may be understood through the influence of mechanical constraints on movement strategy. At the initiation of the deadlift, the bar is positioned anterior to the hip joint, typically resulting in a larger hip moment arm relative to the squat. This configuration increases torque demand on the hip and trunk extensors. More generally, horizontal distance between the body and external load has been associated with low-back moment demands during lifting (12). As the lift progresses, adjustments in trunk position and hip displacement may reduce the hip moment arm, allowing torque demands to be redistributed.

As load approaches maximal levels, these mechanical constraints may not only influence the magnitude of torque demands, but also the strategy used to manage them. Rather than maintaining a fixed coordination pattern, lifters may exhibit joint sequencing patterns consistent with attempts to reduce unfavorable moment arms as the lift progresses. For example, earlier knee extension may be associated with movement patterns in which the hips move closer to the bar, thereby shortening the hip moment arm and reducing torque demand at the hip.

In this way, the sequential characteristics observed in the deadlift may be consistent with a movement strategy shaped by changing mechanical constraints. Changes in segment position may alter leverage conditions as the lift progresses. Such observations suggest that movement under maximal load may not represent a simple scaled version of submaximal technique. As torque demands increase at specific joint angles, maintaining the same coordination pattern may become increasingly difficult, and shifts in joint contribution may emerge as leverage conditions change. Recent computational models provide theoretical support for this possibility by demonstrating that constraints on joint-moment capacity can produce load-dependent changes in posture and redistribution of mechanical demand across joints (9, 10).

## Influence of stance width on mechanical strategy

Stance width is often proposed as a factor that may influence the mechanical relationship between the squat and deadlift. In practice, variations such as wider or semi-sumo deadlift stances are frequently adopted in an effort to create movement patterns that more closely resemble the squat. Changes in stance width can meaningfully alter system geometry. A wider stance may reduce forward trunk inclination and modify hip moment arm demands, potentially changing how torque is distributed across the lower extremity joints. These adjustments can influence both joint loading and perceived effort during the lift.

However, stance width alone does not appear sufficient to override the fundamental mechanical constraints imposed by maximal loading. Regardless of stance, the deadlift is initiated from a static position without an eccentric preload, requiring the lifter to overcome inertia before momentum is established. This initial condition imposes constraints on force production that are not present in the squat. As a result, while stance modifications may influence geometry, they do not fully remove the potential for changes in movement strategy as load approaches maximal levels. Sequential joint contributions and posture adjustments may still emerge as strategies to manage torque demands during the ascent.

These observations suggest that stance width may modulate, but not fundamentally redefine, the mechanical behavior of the deadlift under maximal load. This distinction may help explain why stance modifications can influence perceived similarity between lifts without eliminating underlying differences in how force is produced and distributed under maximal conditions.

## Sticking region considerations

One practical manifestation of these mechanical differences may be observed in the location and nature of the sticking region in each lift. The sticking region is typically defined as the portion of the lift where bar velocity decreases or momentarily stalls despite continued effort. In the squat, the sticking region has been consistently identified at specific joint angles where both hip and knee extensors are required to produce high levels of torque simultaneously (1). Because the movement tends to remain coordinated, this region may reflect a shared mechanical limitation across joints.

In contrast, observations from maximal deadlifting suggest that reductions in bar velocity may occur earlier in the ascent, often before the bar reaches knee height. This may be consistent with an initial mechanical disadvantage associated with a larger hip moment arm and the absence of an eccentric preload. As a result, the deadlift may not exhibit a single, clearly defined sticking point, but rather a transition phase in which joint contributions shift to accommodate changing leverage conditions.

These differences suggest that sticking regions are not solely determined by muscle strength, but also by how mechanical constraints influence coordination patterns under load. As such, the location and nature of the sticking region may differ between lifts as a function of both joint mechanics and movement strategy. These distinctions further support the interpretation that the squat and deadlift may rely on different coordination strategies under maximal load, rather than representing mechanically equivalent applications of lower-body strength.

## Discussion

If squat and deadlift mechanics diverge under maximal load, then the assumption of direct transfer between the lifts warrants reconsideration. While general strength development may improve both lifts during early stages of training, performance at higher levels may be influenced more strongly by lift-specific mechanical constraints. This may help explain why athletes with strong squat performance do not always demonstrate equivalent deadlift performance, and vice versa. Rather than viewing the lifts as mechanically interchangeable, it is more appropriate to consider them as related movements governed by distinct mechanical constraints under high-load conditions. This does not preclude some degree of transfer, particularly during earlier stages of training, but suggests that such transfer may diminish as performance becomes increasingly constrained by lift-specific mechanical demands.

From a practical standpoint, these observations support the principle of specificity in strength training. Developing maximal performance may be influenced by exposure to the unique mechanical demands of each lift, rather than relying solely on improvements in related exercises. Evidence from other performance contexts similarly suggests that relationships between maximal strength and performance may depend on the specific demands of the task or sport (13). In this context, training approaches that treat the squat and deadlift as mechanically interchangeable may overlook important differences in how force is produced and managed under maximal loading conditions.

More broadly, these observations highlight the importance of examining resistance exercises under loading conditions that reflect real-world performance. Studies conducted exclusively at submaximal intensities may not fully capture the movement strategies employed at maximal effort, particularly when coordination patterns are shaped by changing leverage conditions and increasing torque demands. As a result, interpretations derived from submaximal analyses may not fully generalize to high-load performance contexts.

Future research may benefit from further exploration of how movement coordination and joint contributions evolve across loading intensities, particularly in multi-joint resistance exercises. Approaches incorporating competition-based data, higher-intensity protocols, or longitudinal tracking of lifting strategy may provide additional insight into how mechanical constraints are associated with movement organization as loads approach maximal effort.

## Conclusion

The squat and deadlift share important anatomical and functional characteristics, but these similarities do not imply mechanical equivalence under maximal load. Observations from competition-based lifting suggest that each lift is governed by distinct constraints that are associated with differences in movement strategy as force demands increase. Rather than representing interchangeable applications of lower-body strength, the lifts appear to exhibit different movement strategies under high-load mechanical demands.

Recognizing these differences may improve both the interpretation of biomechanical research and the application of training principles in strength development. These observations reinforce the importance of evaluating resistance exercise mechanics under conditions that reflect true performance demands, particularly when informing biomechanical interpretation and strength training practice.
